# Natural product HTP screening for antibacterial (*E.coli 0157:H7*) and anti-inflammatory agents in (LPS from *E. coli O111:B4*) activated macrophages and microglial cells; focus on sepsis

**DOI:** 10.1186/s12906-016-1429-x

**Published:** 2016-11-15

**Authors:** Elizabeth A. Mazzio, Nan Li, David Bauer, Patricia Mendonca, Equar Taka, Mohammed Darb, Leeshawn Thomas, Henry Williams, Karam F. A. Soliman

**Affiliations:** 1 College of Pharmacy & Pharmaceutical Sciences, Florida A&M University, Room 104 Dyson Pharmacy Building, 1520 ML King Blvd, Tallahassee, FL 32307 USA; 2 School of Environment, Florida A&M University, Tallahassee, FL 32307 USA; 3 College of Science & Technology, Florida A&M University, Tallahassee, FL 32307 USA

## Abstract

**Background:**

Acute systemic inflammatory response syndrome arising from infection can lead to multiple organ failure and death, with greater susceptibility occurring in immunocompromised individuals. Moreover, sub-acute chronic inflammation is a contributor to the pathology of diverse degenerative diseases (Parkinson’s disease, Alzheimer’s disease and arthritis). Given the known limitations in Western medicine to treat a broad range of inflammatory related illness as well as the emergence of antibiotic resistance, there is a renewed interest in complementary and alternative medicines (CAMs) to achieve these means.

**Methods:**

A high throughput (HTP) screening of >1400 commonly sold natural products (bulk herbs, cooking spices, teas, leaves, supplement components, nutraceutical food components, fruit and vegetables, rinds, seeds, polyphenolics etc.) was conducted to elucidate anti-inflammatory substances in lipopolysaccharide (LPS) (*E. coli* serotype *O111:B4*) monocytes: RAW 264.7 macrophages [peripheral], BV-2 microglia [brain]) relative to hydrocortisone, dexamethasone and L-N6-(1Iminoethyl)lysine (L-NIL). HTP evaluation was also carried out for lethal kill curves against *E.coli 0157:H7* 1x10^6^ CFU/mL relative to penicillin. Validation studies were performed to assess cytokine profiling using antibody arrays. Findings were corroborated by independent ELISAs and NO2–/iNOS expression quantified using the Griess Reagent and immunocytochemistry, respectively. For robust screening, we developed an in-vitro efficacy paradigm to ensure anti-inflammatory parameters were observed independent of cytotoxicity. This caution was taken given that many plants exert tumoricidal and anti-inflammatory effects at close range through similar signaling pathways, which could lead to false positives.

**Results:**

The data show that activated BV-2 microglia cells (+ LPS 1μg/ml) release >10-fold greater IL-6, MIP1/2, RANTES and nitric oxide (NO2–), where RAW 264.7 macrophages (+ LPS 1μg/ml) produced > 10-fold rise in sTNFR2, MCP-1, IL-6, GCSF, RANTES and NO2–. Data validation studies establish hydrocortisone and dexamethasone as suppressing multiple pro-inflammatory processes, where L-NIL suppressed NO2–, but had no effect on iNOS expression or IL-6. The screening results demonstrate relative few valid hits with anti-inflammatory effects at < 250μg/ml for the following: Bay Leaf (*Laurus nobilis*), Elecampagne Root (*Inula helenium*), Tansy (*Tanacetum vulgare*),Yerba (*Eriodictyon californicum*) and Centipeda (*Centipeda minima*), Ashwagandha (*Withania somnifera*), Feverfew (*Tanacetum parthenium*), Rosemary (*Rosmarinus officinalis*), Turmeric Root (*Curcuma Longa*), Osha Root (*Ligusticum porteri*), Green Tea (*Camellia sinensis*) and constituents: cardamonin, apigenin, quercetin, biochanin A, eupatorin, (-)-epigallocatechin gallate (EGCG) and butein. Natural products lethal against [*E. coli 0157:H7*] where the LC_50_ < 100 μg/ml included bioactive silver hydrosol-Argentyn 23, green tea (its constituents EGCG > Polyphenon 60 > (-)-Gallocatechin > Epicatechin > (+)-Catechin), Grapeseed Extract (*Vitis vinifera*), Chinese Gallnut (its constituents gallic acid > caffeic acid) and gallic acid containing plants such as Babul Chall Bark (*Acacia Arabica*), Arjun (*Terminalia Arjuna*) and Bayberry Root Bark (*Morella Cerifera*)**.**

**Conclusions:**

These findings emphasize and validate the previous work of others and identify the most effective CAM anti-inflammatory, antibacterial compounds using these models. Future work will be required to evaluate potential combination strategies for long-term use to prevent chronic inflammation and possibly lower the risk of sepsis in immunocompromised at risk populations.

## Background

Global health initiatives are encumbered by a vast majority of the population suffering from non-communicable inflammatory diseases such as cardiovascular disease, neurodegeneration, diabetes, arthritis, ulcerative colitis/bowel disease and cancer. Also, with increased incidence of antibiotic resistance, acute inflammation from sepsis plays a major role in mortalities arising from diverse infectious agents [1, 2]. Given limitations in Western medicine to treat/prevent a broad range of inflammatory related illness, there is a renewed interest in complementary and alternative medicines (CAMs) to achieve these means [3–9].

While there exist a plethora of scientific publications on the efficacy of individual CAMs in specific inflammatory models, there lacks a relative comparative potency rank of the most commonly marketed CAMs in a single study, conducted under uniform conditions. Our high throughput (HTP) screening library houses over 1400 products most which are available and sold to consumers throughout the world in the form of bulk herbs, cooking spices, teas, leaves, supplement components, nutraceutical food components, fruit and vegetables, roots, rinds, seeds, polyphenolics etc. The purpose of the current study is to screen commonly utilized CAMs for anti-inflammatory efficacy under uniform standard conditions to elucidate the most potent at non-toxic/low therapeutic concentrations (<250 μg/mL), and further to compare these to steroidal and NSAID drugs.

The in-vitro model employed was that of monocytes (peripheral and central nervous system) stimulated by lipopolysaccharide (LPS) derived from *E. coli O111:B4.* LPS is a cell wall endotoxic component from gram negative bacteria which evokes a deadly cytokine storm associated with septicemia, septic shock and multi organ failure. Known biologic consequences of LPS include the colossal release of chemotactic cytokines, IL-3 IL-12, TNF-alpha, IL-6, IL-1 beta, inducible nitric oxide (iNOS) NO_3_ -/NO_2_ -, P-selectin, CD 11b/CD18 (Mac-1) ICAM-1, PGE2 which enable massive neutrophil infiltration and hemolytic [10–13]. While many of these inflammatory molecules at high concentrations are lethal, sub-chronic rises of the same are associated with age related inflammatory degenerative diseases such as Parkinson’s disease, Alzheimer’s disease and arthritis [14–17]. Therefore, the use of LPS in this model and subsequent elucidation of the most effective CAMs against inflammatory parameters, can provide information on potential therapeutics for both chronic and acute inflammatory processes.

In this study, we conduct a HTP screening of CAMs to assess both capacity to kill a pathogenic strain of *E.coli 0157:H7* as well as to mitigate the pro-inflammatory effects from *E.Coli* derived endotoxin cell wall component; LPS.

## Methods

Hanks Balanced Salt Solution, (4-(2-hydroxyethyl)-1-piperazineethanesulfonic acid) (HEPES), ethanol, sulfanilamide, 96 well plates, general reagents and supplies, were all purchased from Sigma-Aldrich, (St Louis, MO, USA) or VWR (Radnor, PA, USA). Imaging probes were purchased from Life Technologies (Grand Island, NY, USA). Natural products were purchased from Frontier Natural Products Co-op (Norway, IA, USA), Monterey Bay Spice Company (Watsonville, CA, USA), Mountain Rose Herbs (Eugene, OR, USA), Mayway Traditional Chinese Herbs (Oakland, CA, USA), Kalyx Natural Marketplace (Camden, NY, USA), Futureceuticals (Momence, IL, USA), organic fruit vegetable market: New Leaf (Tallahassee, FL, USA), Florida Food Products Inc. (Eustis, FL, USA), Patel Brothers Indian Grocery (Tampa, FL, USA), Opil Gold from Aging Sciences LLC (Wayland, MA, USA) and Colloidal Silver - Argentyn 23® Natural Immunogenics (Sarasota, FL, USA). Elisa kits and cytokine antibody arrays were purchased from Assay Biotech (Sunnyvale, CA) and Raybiotech (Norcross, GA, USA).

### Cell culture

BV-2 microglia (BV-2) cells were provided by Elizabeta Blasi [18], and RAW 264.7 cells were purchased from American Type Culture Collection (Manassas, VA, USA). Cells were cultured in DMEM high glucose media [glucose 4500 mg/L] containing 5% FBS, 4 mM L-glutamine, and penicillin/streptomycin (100 U/0.1 mg/mL). Culture conditions were maintained at 37 °C in 5% CO_2_/atmosphere and every 2–3 days, the media was replaced and cells sub-cultured. For experiments, plating media consisted of DMEM (minus phenol red) [glucose 4500 mg/L], 2.5% FBS and penicillin/streptomycin (100 U/0.1 mg/mL). LPS O111:B4 was prepared in HBSS at 1 mg/mL and stored at –20 °C. For experiments, LPS was added to the culture media at a working concentration of 1μg/mL.

### Bacterial culture

A single colony of *E. coli O157:H7* was grown on an agar plate. *E. coli* was then inoculated into a 20 mL of Luria-Bertani (LB) in a flask, grown at 37 °C with moderate shaking (180 rpm), to an OD 600 *nm* = 0.6. One mL of the culture suspension was moved into a 1.5 mL Eppendorf tube and centrifuged for 1 min at 10,000 g (4 °C). After discarding the supernatant, the bacterial pellet was re- suspended in 1 mL sterilized water. This centrifugation was repeated twice. The bacteria were stored at 4 °C. The bacterial cell numbers were then determined using colony forming units (CFU) through serial dilution plating on LB plate at 37 °C. The experimental concentration of *E. coli* was 1 x 10^6^ CFU/mL.

### Sample preparation

All natural chemicals and reference drugs were dissolved in DMSO [5–20mg/mL] and crude herbs in absolute ethanol [50 mg/mL] after being diced, macerated and powdered then stored at –20 °C. All plants were cataloged by manufacturer, botanical and common names. All dilutions were prepared in sterile HBSS + 5 mM HEPES, adjusted to a pH of 7.4, ensuring solvent concentration of DMSO or absolute ethanol at less than 0.5%.

### Cell and microbial -viability

Cell and microbial viability were assessed using resazurin [7-Hydroxy-3H-phenoxazin-3-one 10-oxide] (Alamar Blue) indicator dye [19]. A working solution of resazurin was prepared in sterile HBSS minus phenol red (0.5 mg/mL), then added (15% v/v) to each sample. Samples were returned to the incubator for 2–4 h, and reduction of the dye by viable cells (to resorufin, a fluorescent compound) was quantitatively analyzed using a Synergy HTX multi-mode reader Bio-Tek Inc. (Winooski, VT, USA) with settings at [550 *nm*/580 *nm*], [excitation/emission].

### In-Vitro efficacy index

Several methodological concerns were addressed regarding HTP screenings. These included basic controls for pH (neutralized with buffered HBSS) and cell viability. In-vitro, immortal (malignant) immunocompetent cell lines such as glioma cells, macrophages, microglia, lymphocytes or granulocytes are of tumor origin, and many natural compounds simultaneously induce apoptosis in malignant cells and attenuate inflammation via the same pathways (i.e. phosphorylation of extracellular signal-regulated kinase (ERK), c-jun NH2-terminal kinase (JNK) phosphorylation and mitogen-activated protein kinases (MAPK)/NF-κB) [20–26]. For this reason, we constructed and utilized an in-vitro efficacy index (*i*EI) paradigm, to ensure that anti-inflammatory effects are occurring at non-cytotoxic concentrations. The *i*EI is defined as the LC_50_ (toxic concentration)/IC_50_ (anti-inflammatory concentration) ratio, with higher values reflecting a greater confidence in the anti-inflammatory effects occurring independently of cell death.

### Nitrite (NO_2_–)/iNOS expression

Quantification of nitrite (NO_2_−) was determined using the Greiss reagent [27]. The Greiss reagent was prepared by mixing an equal volume of 1.0% sulfanilamide in 10% phosphoric acid and 0.1% N-(1-naphthyl)-ethylenediamine in deionized water, which was added directly to the cell supernatant (experimental media consisting of DMEM - phenol red) and incubated at room temperature for 10 min. Controls and blanks were run simultaneously, and subtracted from the final value to eliminate interference. Samples were analyzed at 540 nm on a Synergy HTX multi-mode reader; Bio-Tek (Winooski, VT, USA).

iNOS protein expression was determined using immunocytochemistry. Cells were fixed in 4% paraformaldehyde/permeabilized in 0.2% Triton X 100 in phosphate buffered saline (PBS) and incubated with anti-iNOS, an N-Terminal antibody produced in rabbit for 24 h at 4 °C in a casein blocking buffer. Samples were washed in PBS, then incubated with anti-rabbit Alexa Fluor® 488 conjugate for two hours at RT. Samples were counterstained with propidium iodide and imaged using a fluorescent/inverted microscope, CCD camera and data acquisition using ToupTek View ; ToupTek Photonics Co (Zhejiang, P.R.China).

### Mouse cytokine antibody array

Mouse Cytokine Antibody Arrays (Product Code: AAM-CYT-1000) Ray Biotech; (Norcross, GA, USA) were used to profile the effects of LPS (1μg/mL) on BV-2 and RAW 234.7 cell lines. Each experiment was carried out according to the manufacturer’s instructions, and in triplicate. Briefly, antibody-coated array membranes were first incubated for 30 min with 1 mL of blocking buffer. After 30 min, blocking buffer was decanted and replaced with 1 mL supernatant from control (untreated) samples, cells treated with (1ug/mL LPS only) and a media blank. Membranes were incubated overnight at 4 °C with mild shaking. The next day, the medium was decanted; membranes were washed, and subsequently incubated with 1 mL biotin-conjugated antibodies for 6 h. Lastly, biotin-conjugated antibodies were removed and membranes were incubated with HRP-conjugated streptavidin (2h), then evaluated for densitometry using a chemiluminescence substrate monitored on a VersaDoc Imager/Quantity One software from Bio-Rad: (Hercules, CA, USA).

### IL-6 (Interleukin-6) ELISA

After experimental treatment, cells supernatants were directly evaluated for concentration of IL-6 using a Murine OmniKine™ IL-6 ELISA (Product Code # OK-0187), Assay Biotechnology Inc. (Sunnyvale, CA, USA), performed according to the manufacturer’s guidelines. Data was quantified by optical density at 450 *nm* using a Synergy HTX multi-mode reader from Bio-Tek (Winooski, VT, USA).

### Data analysis

Statistical analysis was performed using Graph Pad Prism (version 3.0; Graph Pad Software Inc. San Diego, CA, USA) with significance of difference between the groups assessed using a one-way ANOVA then followed by Tukey post hoc means comparison test, or a Student’s t test. IC_50_s were determined by regression analysis using Origin Software (Origin Lab, Northampton, MA).

## Results

### Validation

**Fig. 1:**
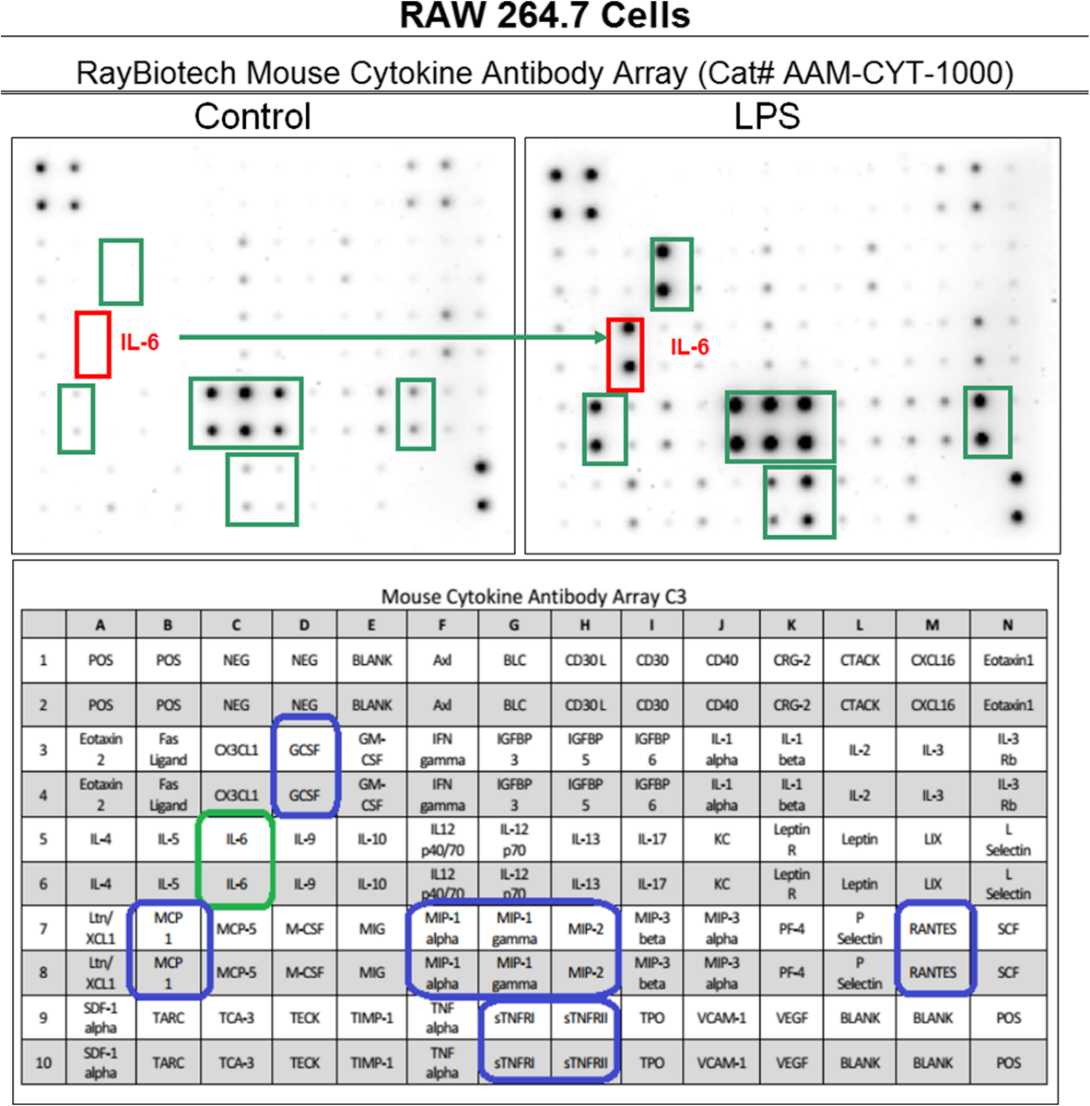
Cytokine release profile in LPS activated RAW 264.7 cells. The data are displayed as the cytokine array blot image and array grid layout with leading changes presented in highlighted boxes

**Fig. 2:**
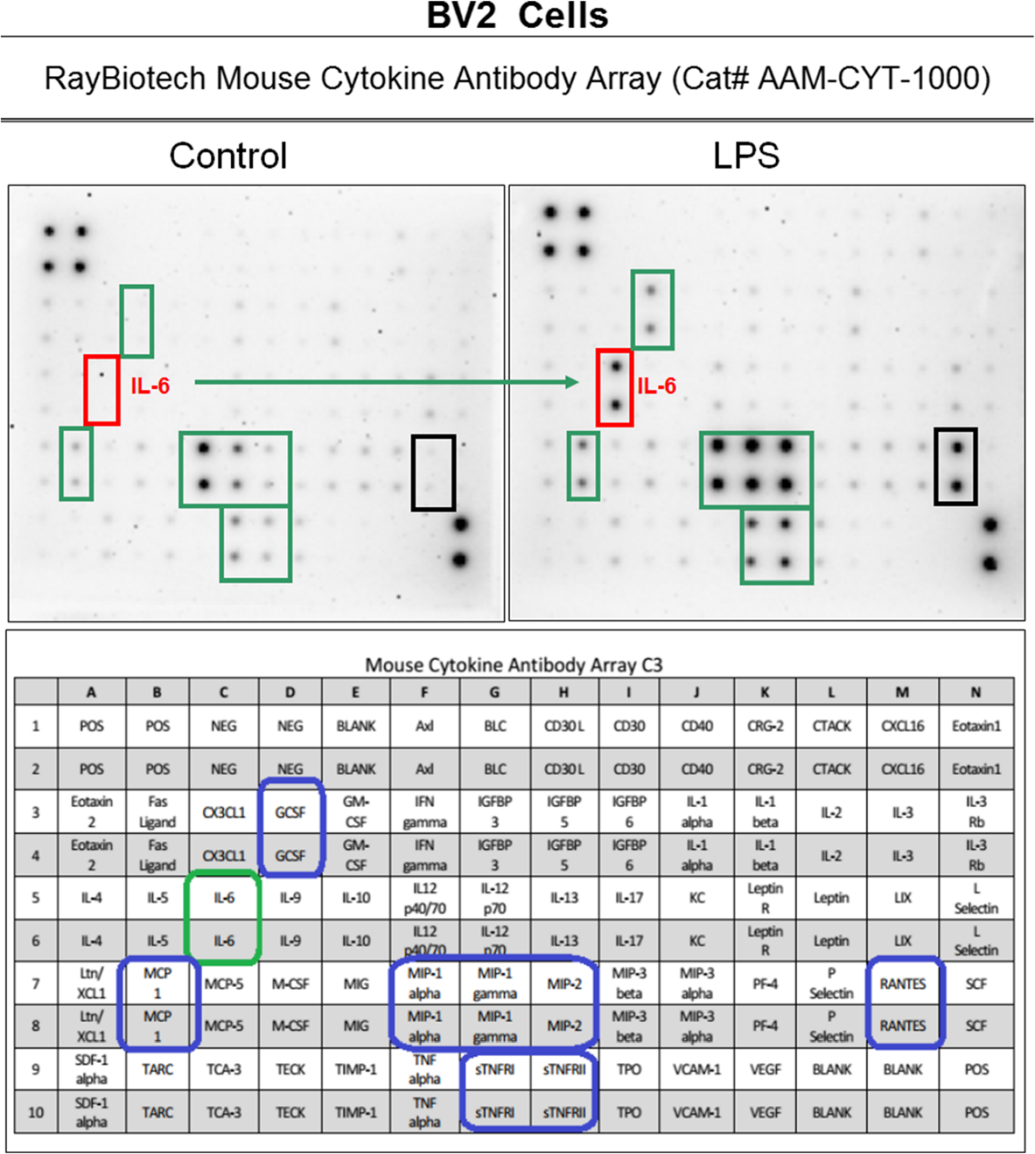
Cytokine release profile in LPS activated BV-2 cells. The data are displayed as the cytokine array blot image and array grid layout with leading changes presented in highlighted boxes

**Fig. 3:**
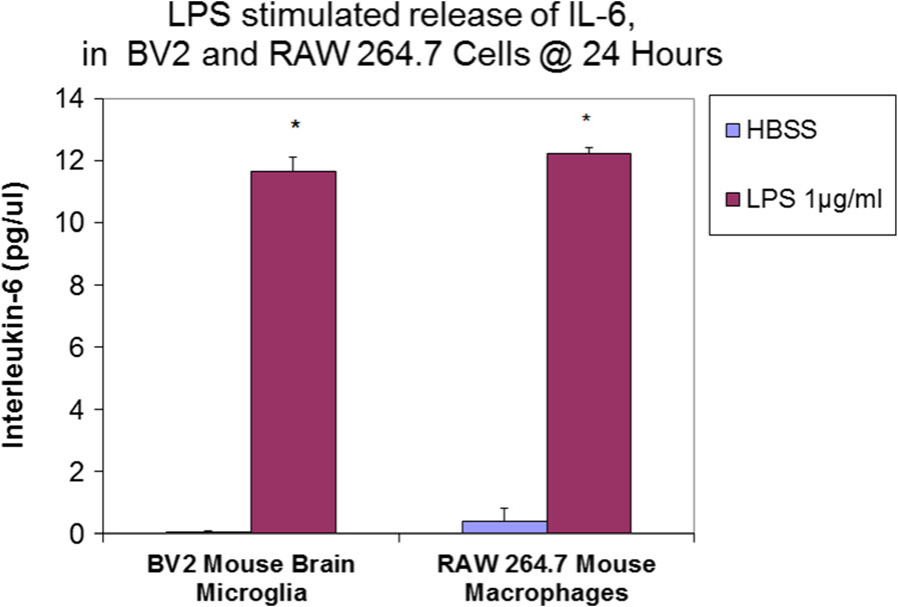
ELISA: Quantification of IL-6 in the supernatant of resting and LPS activated cells (BV-2 cells, RAW 264.7 cells). The data represent IL-6 *(pg/μl)* and are expressed as the Mean ± S. E. M., *n* = 3. Differences between resting and LPS activated cells were determined by a student’s T test (*) *P* < 0.001

**Fig. 4:**
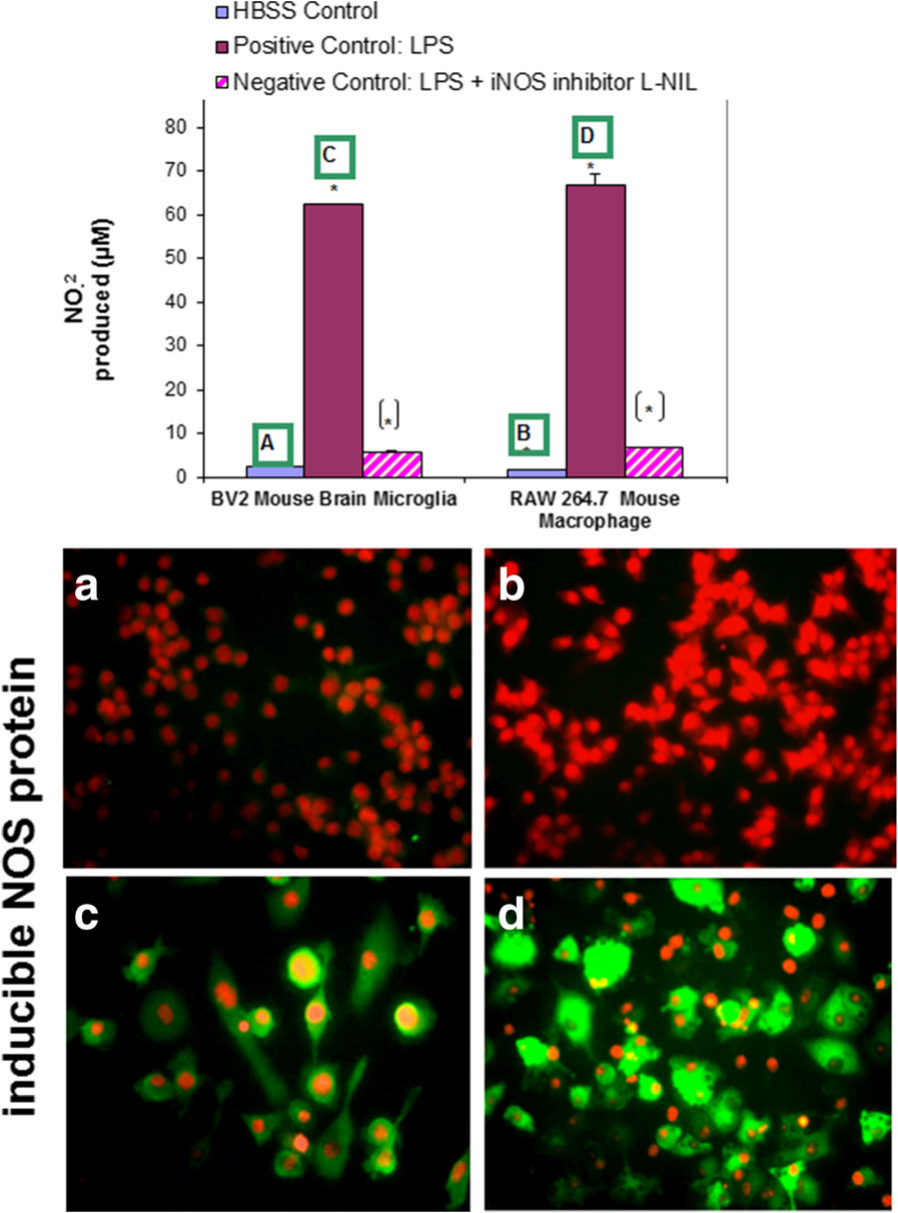
NO2- production in resting and LPS activated cells (BV-2 cells, RAW 264.7 cells) ± selective iNOS inhibitor: L-NIL (12 μg/mL). The data represent NO2- produced (μM) and are expressed as the Mean ± S. E. M., *n* = 3. Differences between resting and LPS activated cells were determined by a student’s T test (*) *P* < 0.001. Differences between LPS vs. LPS/L-NIL treated cells were determined by a student’s T test [*] *P* < 0.001. iNOS expression was analyzed by ICC using rabbit anti-mouse iNOS/goat anti-rabbit Alexafluor 488, in fixed permeabilized, propidium iodide nuclear counterstained cells (**a**) resting BV-2 cells (**b**) resting RAW 264.7 cells (**c**) LPS activated BV-2 cells (**d**) and RAW 264.7 cells

Validation studies were conducted to determine profiled cytokine differentials in LPS activated RAW 264.7 (Fig. 1) and BV-2 cells (Fig. 2), respectively - using semi quantitative antibody microarrays, which were run in triplicate. The representative panel shows both cell lines exposed to LPS prompted the greater release of MCP-1, GCSF, MIP1a, MIP1g and MIP-2, sTNFR1/11, RANTES and IL-6. Quantitative analysis of IL-6 was corroborated by ELISA (Fig. 3), and iNOS protein expression was evaluated by ICC (Fig. 4b) and NO2- release using the Griess Reagent (Fig. 4a), the latter of which was reduced in the presence of iNOS inhibitor (L-NIL).

### Screening

**Fig. 5:**
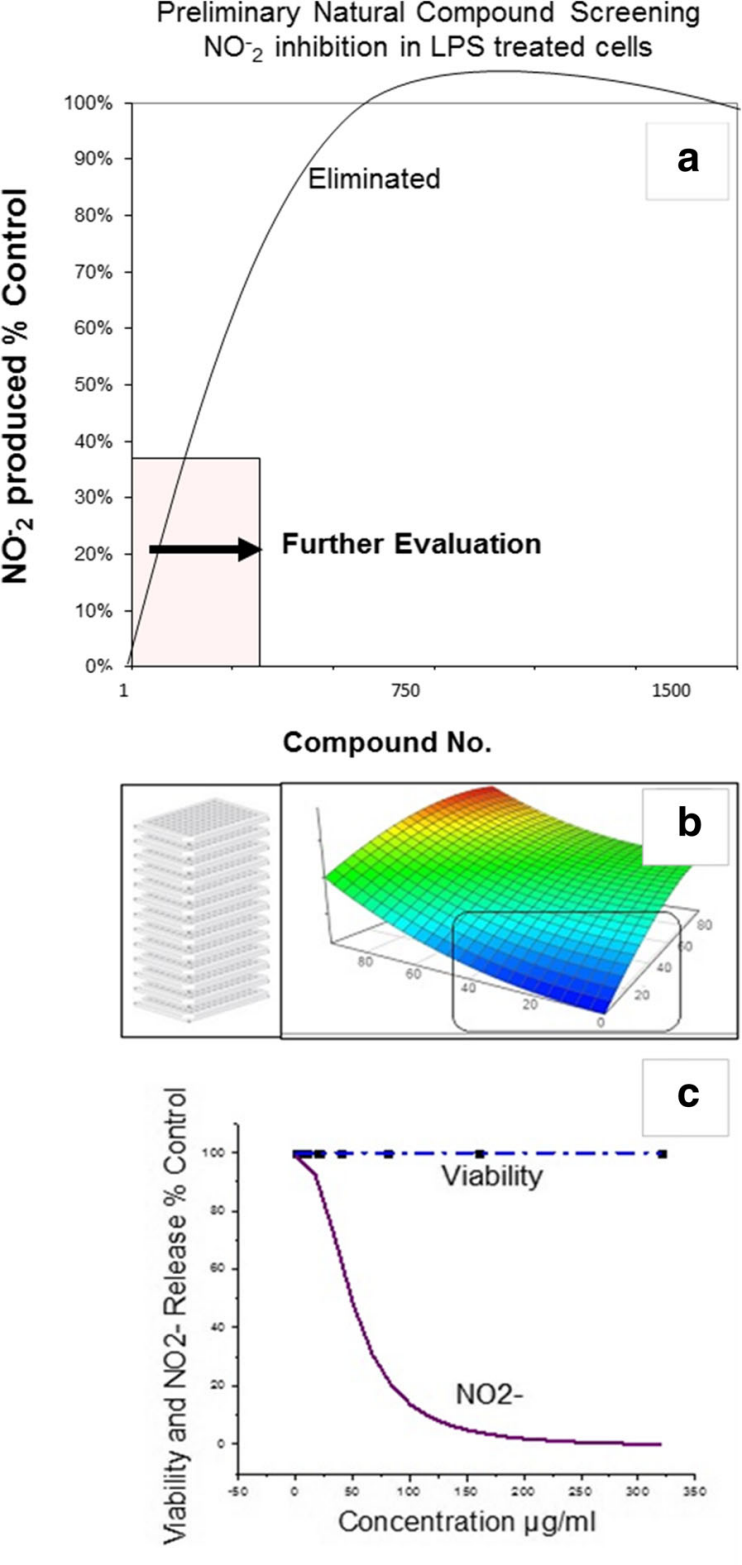
High-through-put study design. The basic study layout consisted of a primary first level tier 1 screening by which all CAMs were tested to reduce NO_2_- in LPS treated BV-2 and RAW 264.7 cells [maximum working concentrations were : 230μg/mL (plant based) and 92μg/ml (metabolites, drugs and polyphenolics)]. **a**, **b** Compounds displaying an IC_50_ below the 1st tier concentrations were further evaluated as per the template (**c**). All compounds were simultaneously evaluated for toxicity/anti-inflammatory effects and an *i*EI differential was established (LC_50_/IC_50_) to prevent false positives incurred by cytotoxic effects

**Fig. 6:**
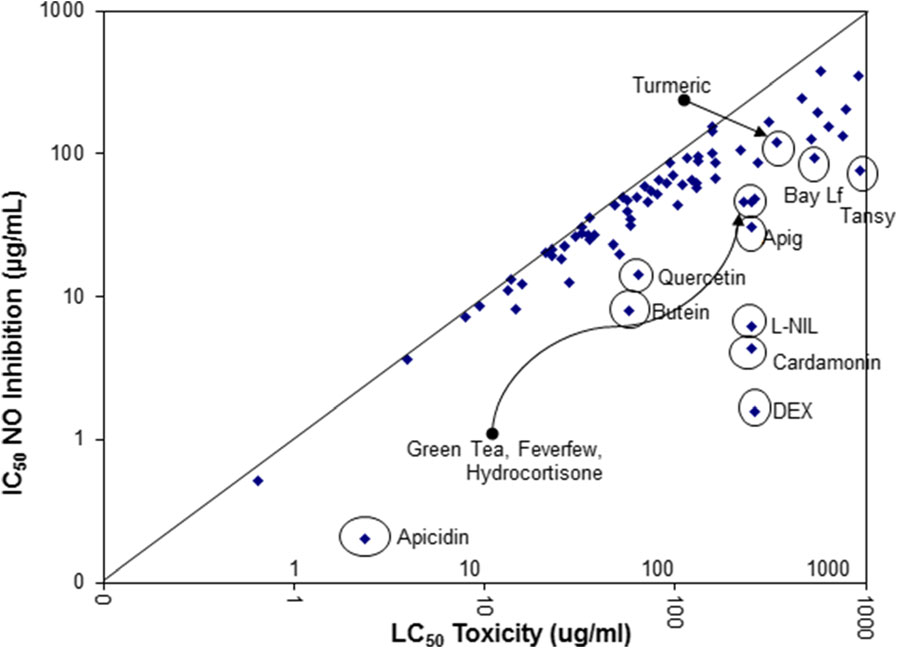
In-vitro efficacy scatter-plot for NO_2_–, inhibition vs. cell toxicity in RAW 264.7 cells. The data are presented as LC_50_ (toxicity) vs. IC_50_ (iNOS inhibition) determined from a regression analysis on a minimum of 6 concentrations

**Fig. 7:**
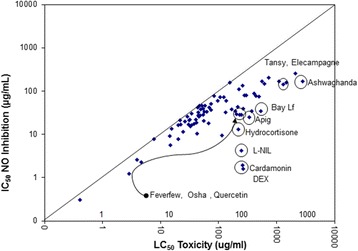
In-vitro efficacy scatter-plot for NO_2_–, inhibition vs. cell toxicity in BV-2 cells. The data are presented as LC_50_ (toxicity) vs. IC_50_ (iNOS inhibition) determined from a regression analysis on a minimum of 6 concentrations

**Fig. 8:**
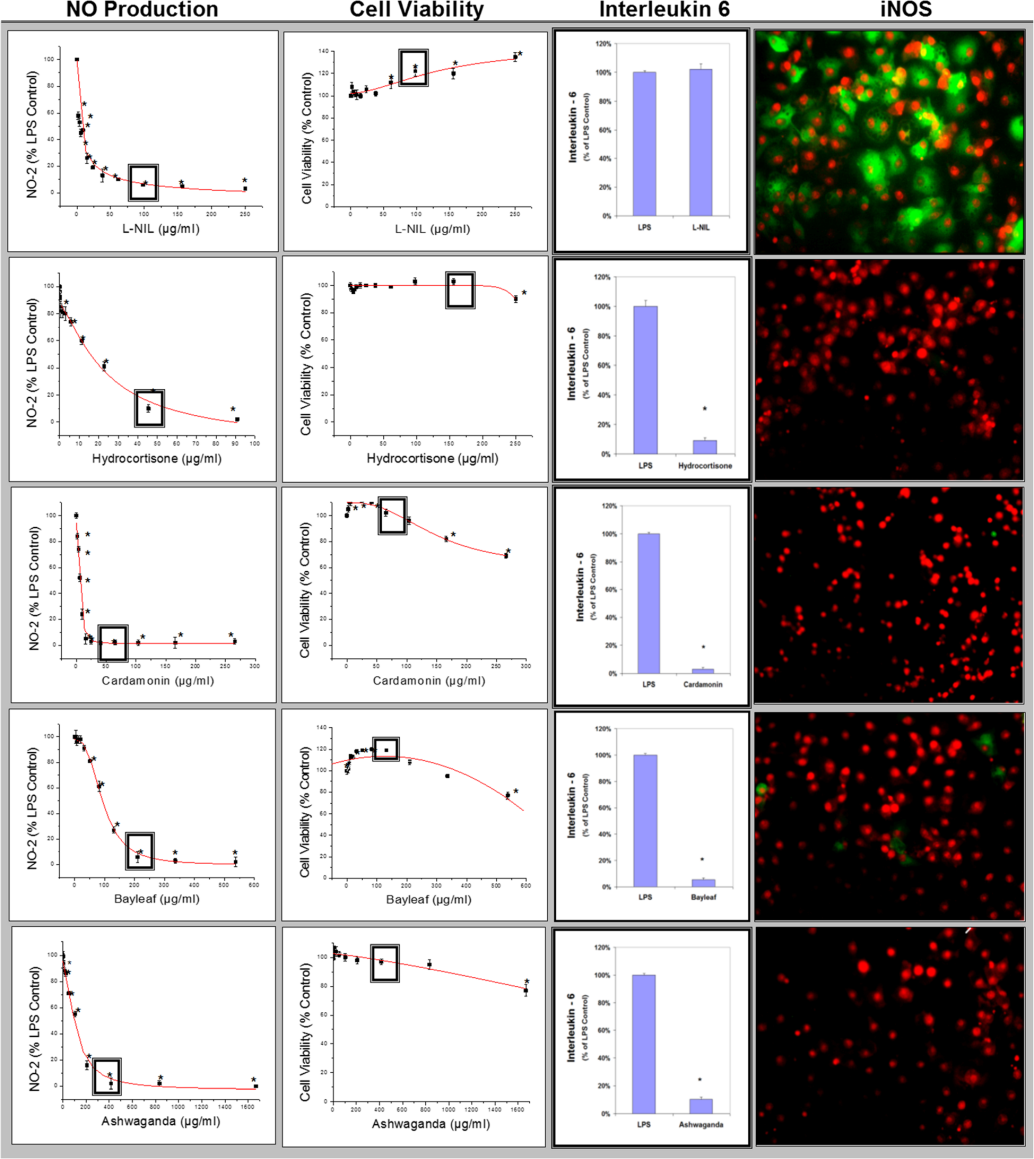
Linear regression profiles for NO2- inhibition and toxicity in RAW 264.7 cells, by which LC_50s and_ IC_50_s were calculated for all compounds, presented along with corresponding iNOS ICC images and IL-6 release measured at concentrations reflected by a square (◘). The data represent NO2- and viability (% LPS Control), presented as the Mean ± S.E.M, *n* = 4. Statistical difference from the Controls were determined by a one-way ANOVA, followed by a Tukey post – hoc test * *P* < 0.05 and IL-6 from controls by a student’s t-test* *P* < 0.05

**Fig. 9:**
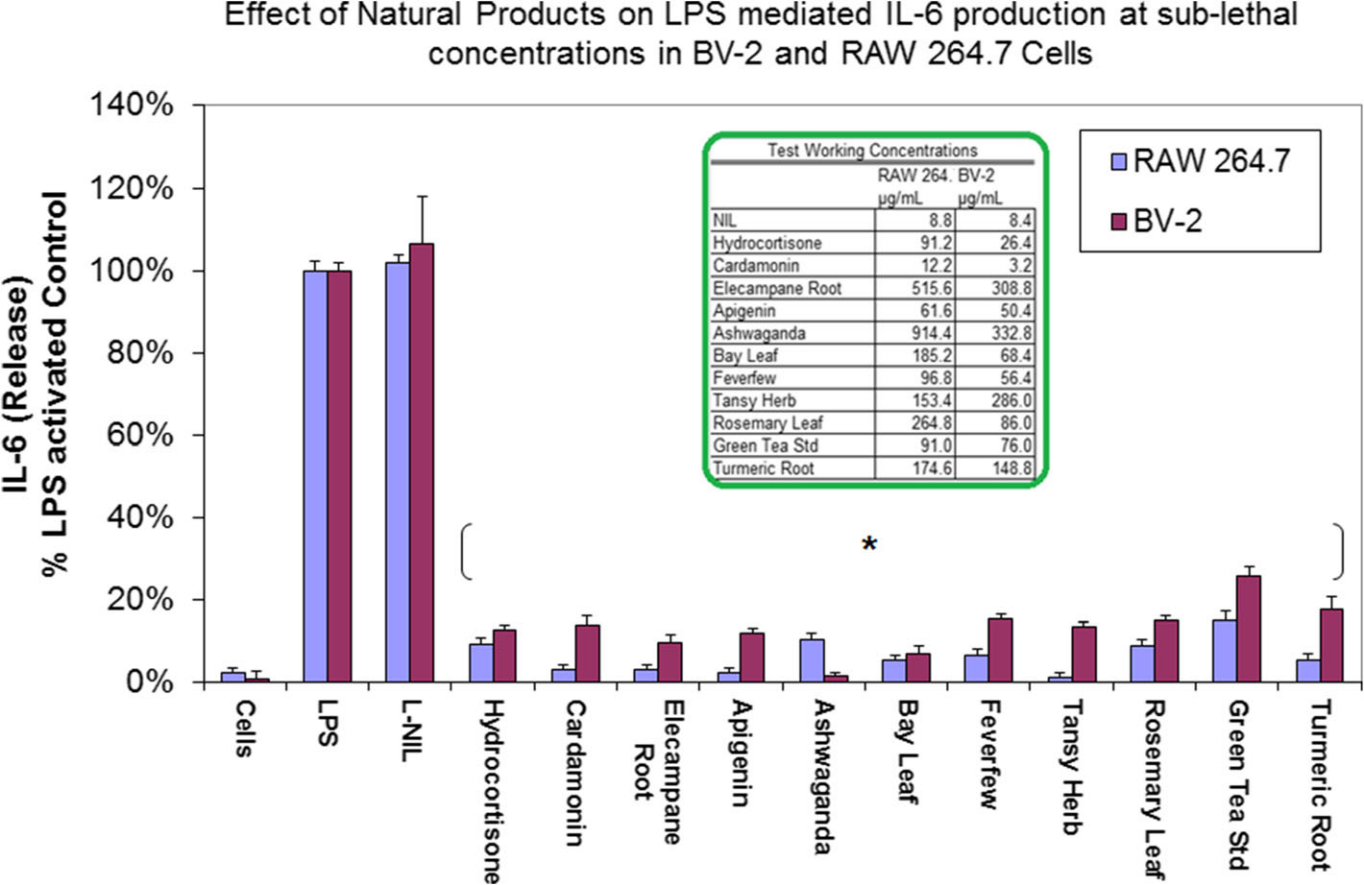
Effects of lead anti-inflammatory products on IL-6 release in LPS treated BV-2 and RAW 264.7 cells. The data represents IL-6 (as % LPS Control) and expressed as the Mean ± S.E.M., *n* = 3. Differences between activated cells ± natural compounds at sub-lethal dose were determined by a student’s T test (*) *P* < 0.001

**Fig. 10:**
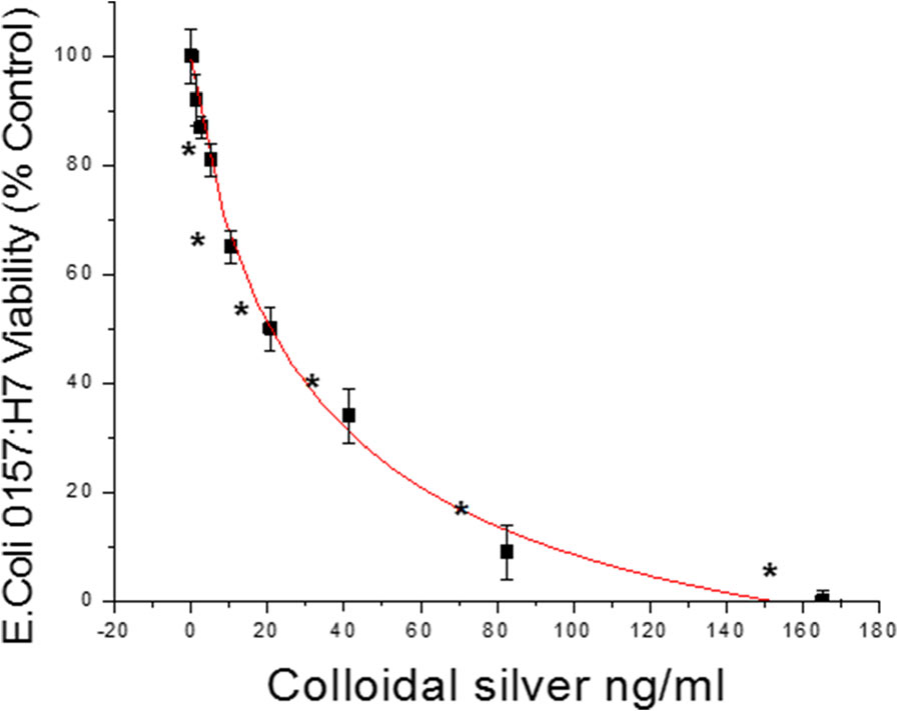
The effect of colloidal Silver-Argentyn 23® on the survival of E.coli 0157:H7 (1x10^6^ CFU/ml) @ 8 h in 31°C. The data represent viability (% Control), presented as the Mean ± S.E.M, *n* = 4. Statistical differences from the Control were determined by a one-way ANOVA, using the Tukey post – hoc test. * *P* < 0.05

The initial HTP screening was conducted using a natural plant library housing over 1400 extracts including: 1) Plants: seeds, fruits, vegetables and herbs (of diverse ethnic nature including Chinese, Egyptian, Indian etc.) 2) Natural derived chemicals/polyphenolics 3) Metabolic Substrates: amino acids, vitamins and energy intermediates such as organic acids, glycolytic intermediates and 4) Reference NSAID and steroidal anti-inflammatory drugs. The preliminary screen was conducted to assess reduction of NO2- in LPS activated cells [equal to or less than 230 μg/mL] for all compounds (Fig. 5a). Substances that attenuated NO_2_– at less than 50% of the 1^st^ tier starting concentrations, were re-evaluated over a dose range where LC_50s_ (cytotoxicity) and IC_50s_ (NO2-) were simultaneously evaluated (Fig. 5b, c). From the linear regression, LC_50_, IC_50s_ concentrations were determined and in-vitro efficacy index (iEI) was calculated by the ratio value : LC_50_/IC_50_ . The higher the ratio, the greater confidence of true anti-inflammatory effects, not attributable to cell death. All *i*EI values are presented in Table 1 for RAW 264.7 cells/Table 2 for BV-2 cells, with matching logarithmic scatter-plots (Figs.6 and 7). Figure 8 shows a sample of NO2-/viability dose response data, with corresponding immunochemical imaging for iNOS in RAW 264.7 cells, where supernatant was evaluated for IL–6. The data from these experiments show that L-NIL, while capable of inhibiting the catalytic function of iNOS, was not an anti-inflammatory in the true sense. L-NIL suppressed NO_2_- but had no effects on cytokine release or expression of iNOS. Most lead compounds that reduced NO2- in both cell lines at sub-lethal concentrations (2 x IC_50_ for NO2-inhibition) which corresponded to a reduction of IL-6 in sample supernatant (Fig. 9). The antimicrobial effects of natural products on the survival of E.coli 0157:H7 (1x10^6^ CFU/mL) were then evaluated. The data show only a select few have therapeutic potency relative to penicillin/streptomycin (Table 3), colloidal silver being the most effective (Fig. 10). The findings from this study delineate the most potent anti-inflammatory/and antibacterial natural compounds, when conducted in a uniform controlled fashion in these particular models.

**Table 1:** Efficacy of anti-inflammatory CAMS relative to cellular toxicity in LPS activated RAW 264.7 cells

RAW 264.7 cells + 1μ/mL LPS: anti-inflammatory potency			
Substance	Anti-inflammatory IC_50_ (μg/mL)	Toxicity LC_50_ (μg/mL)	*i*EI (LC_50_/IC_50_)
L-N-lysine dihydrochloride	4.4	250.0	*>*57.4
Cardamonin	6.1	250.0	>40.8
Dexamethasone	1.6	260.0	>22.4
Hydrocortisone	45.6	250.0	*>*5.4
Bay Leaf/*Laurus nobilis*	92.6	537.0	*>*5.8
Tansy Herb/*Tanacetum vulgare*	76.7	934.9	12.2
Apicidin	0.2	2.4	11.7
Apigenin	30.8	252.0	8.2
Yerba Santa Lf/*E. californicum*	194.0	1413.4	7.3
Butein	8.0	57.9	7.3
Ashwagandha/*Withania somnifera*	457.2	3306.0	7.2
Centipeda Herb/*Centipeda minima*	213.7	1260.5	5.9
Rosemary Lf/*Rosmarinus officinalis*	132.4	754.8	5.7
Feverfew/*Tanacetum parthenium*	48.4	264.2	5.5
Green Tea Std sigma T5550	45.5	228.7	5.0
Elecampane Root/*Inula helenium*	257.8	1276.0	4.9
Quercetin	14.3	63.8	4.5
Commiphora myrrha resin	127.1	5221.1	4.1
Amla/*Phyllanthus emblica*	156.7	641.0	4.1
Herb de province	203.4	793.2	3.9
Turmeric Root/*Curcuma longa*	87.3	274.3	3.1
Biochanin A	119.0	345.1	2.9
Trifala	195.3	559.5	2.9
Cinnamon/*Cinnamomum burmannii*	344.9	923.0	>2.6
EGCG	20.0	50.8	2.5
Bergamottin	67.1	161.7	2.4
Osha Root/*Ligusticum porteri*	43.4	104.1	2.4
Kalijiri Purple Fleablame	58.0	130.0	2.2
Curcumin	12.6	28.1	2.2
Rabdosia rubescens Herb	104.9	220.3	2.1
White Sage/*Salvia apiana*	62.0	129.5	2.1
Blood Root/*Sanguinaria canadensis*	23.4	47.4	2.0

The data represent LC_50_ values for toxicity and IC_50_ values for NO2- reduction both determined by regression analysis on a minimum of 6 concentrations,(*n* = 4). The ratio of LC_50_/IC_50_ μg/mL is the *i*EI (*in- vitro* efficacy index), where the greater the value the greater the confidence in the anti-inflammatory effects. The symbol [>] denotes an *i*EI value acquired on a maximum upper limit concentration being tested

**Table 2:** Efficacy of natural anti-inflammatory compounds relative to cellular toxicity in LPS activated BV-2 cells

BV-2 microglia cells + 1μg/mL LPS: anti-inflammatory potency			
Substance	Anti-inflammatory IC_50_ (μg/mL)	Toxicity LC_50_ (μg/mL)	*i*EI (LC_50_/IC_50_)
Cardamonin	1.6	265.0	>169.4
Dexamethasone	1.9	260.0	>136.8
Bay Leaf/*Laurus nobilis*	34.2	537.0	>15.7
Quercetin	27.8	250.0	>8.9
Apicidin	0.0	0.6	65.5
L-N-lysine dihydrochloride	4.2	247.2	58.8
Elecampane Root/*Inula helenium*	154.4	1486.0	35.7
Ashwagandha/Withania somnifera	166.4	2848.3	17.1
Hydrocortisone	13.0	219.2	16.9
Apigenin	25.2	337.0	13.4
Optilgold	9.4	113.6	12.1
Biochanin A	33.7	369.2	10.9
Tansy Herb/*Tanacetum vulgare*	143.0	1302.1	9.1
Feverfew/*Tanacetum parthenium*	28.2	230.2	8.2
Centipeda/*Centipeda minima*	258.4	2105.2	8.1
Osha Root/*Ligusticum porteri*	29.5	203.4	6.9
Eupatorin	39.3	265.2	6.7
Turmeric Root/*Curcuma longa*	74.4	498.9	6.7
Herb de province	167.2	1115.3	6.7
Granati peel/Punica granatum rind	75.7	439.1	5.8
Rabdosia rubescens Herb	34.5	192.7	5.6
Rosemary Lf/*Rosmarinus officinali*	43.0	214.8	5.0
Trifala	88.3	408.8	4.6
Green Tea Std Sigma T5550	38.0	169.7	4.5
Curcumin	10.2	43.2	4.2
Myrrh/*Commiphora myrrha*	31.8	132.8	4.2
Clove/*Syzygium aromaticum*	149.0	615.1	4.1
Indomethacin	17.9	67.2	3.7
Sage leaf/*Salvia officinalis*	80.3	298.4	3.7
Amla/*Phyllanthus emblica*	206.1	736.1	3.6
White Sage/*Salvia apiana*	79.2	282.0	3.6
Ganthoda	168.3	563.7	3.3
Succinum Resin	20.1	56.3	2.8
Genistein	5.6	14.7	2.6
Baicalein	7.8	20.1	2.6
Butein	1.2	2.9	2.3
Maddar root/*Rubia tinctorum*	59.2	135.0	2.3
Yerba Santa Lf/E. *californicum*	18.3	41.4	2.3
EGCG	11.3	24.8	2.2
Phloretin	17.2	37.8	2.2
Frankincense/*Boswellia carterii*	15.4	32.5	2.1
Fisetin	2.3	4.7	2.1
Piperine	36.2	75.1	2.1
Javentri Powder	16.5	34.1	2.1
Bergamottin	111.4	227.5	2.0
Cinnamon/*Cinnamomum burmann*	135.0	260.0	<1.92

The data represent LC_50_ values for toxicity and IC_50_ values for NO2- reduction both determined by regression analysis on a minimum of 6 concentrations,(*n* = 4). The ratio of LC_50_/IC_50_ μg/mL is the *i*EI (*in- vitro* efficacy index), where the greater the value the greater the confidence in the anti-inflammatory effects. The symbol [>] denotes an *i*EI value acquired on a maximum upper limit concentration being tested

**Table 3:** Efficacy of CAM antibacterial compounds on survival of E.coli 0157:H7 (1x10^6^ CFU/ml) @ 8 h in 31 °C

Anti-bacterial effects of natural compounds on E. coli 0157:H7 survival		
Compound	LC_50_	Units
Penicillin/Streptomycin	0.01	Units/μg.ml
Cholloidal Silver-Argentym 23®	0.02	μg/ml
(+) Gossypol	1.5	μg/ml
Grapeseed Extract/*Vitis Vinifera*	1.8	μg/ml
Green Tea Extract/*Camellia Sinensis*	2.0	μg/ml
(-)-Epigallocatechin gallate	3.7	μg/ml
Doxorubin hydrochloride	5.4	μg/ml
Gallic Acid	8.4	μg/ml
Caffeic Acid	10.1	μg/ml
Chinese Gallnut	10.4	μg/ml
Babul Chall Bark/*Acacia arabica*	13.4	μg/ml
Polyphenon 60	14.1	μg/ml
Uva Ursi/*Arctostaphylos uva ursi*	19.1	μg/ml
Arjun/*Terminalia arjuna*	23.8	μg/ml
Balm of Gilead Bud/*Populus candicans*	26.7	μg/ml
Bayberry Root/*Morella cerifera*	28.5	μg/ml
Blood Root/Sanguinaria canadensis/	33.5	μg/ml
(-)-Gallocatechin	35.8	μg/ml
2-D08	37.0	μg/ml
Glyoxal Acid	39.6	μg/ml
Scutellarian	42.0	μg/ml
Kokum Black/*Garcinia Indica*	47.5	μg/ml
Indole	47.5	μg/ml
Trifala	47.7	μg/ml
CraneSbill Root/*Geranium maculatum*	51.6	μg/ml
Shi Liu Pi (Granati Peel)	60.4	μg/ml
Catuaba Bark/*Trichilia Catigua*	63.4	μg/ml
Chapparal/Larrea tridentata	73.6	μg/ml
Thymol	92.0	μg/ml
Esculetin	93.3	μg/ml
Epicatechin	114.0	μg/ml
Piperonal	115.1	μg/ml
(+)-Catechin	173.0	μg/ml

The data represents LC_50_ values determined by regression analysis, on a minimum of 6 concentrations, (*n* = 4)

## Discussion

The data from this study establish several findings including [A] uncorroborated anti-inflammatory/antimicrobial effects for over a thousand natural compounds at concentrations less than 230 μg/mL using this model; [B] corroborating data of existing work by other research groups regarding anti-inflammatory effects of green tea, curcumin, turmeric and rosemary ; and antimicrobial properties of green tea, its catechins, Chinese gallnut, gallic acid plant derived anti-fungal agents (cotton/gossypol) or silver nanoparticle dispersions [28–37]. Lastly; [C] this work provides new evidence on some lesser acknowledged herbs to which historical medicinal value has been attributed, but little research has been documented. Some of these include the following.

Elecampane **(** *Inula helenium*) (IH) has extensive historical medicinal value, where its use dates back to the Iron Age (c. 800–450 B.C.) throughout the third century B.C. to 79 A.D. also mentioned by Pliny and further corroborated in the Chilandar Medical Codex (13th or 14th centuries A.D.) [38]. Within the last century, scientific documentation is somewhat sparse on this herb having primarily focused on its ability to cause allergic dermatitis or act as an anti-cancer agent attributable to the content of alantolactone and isoalantolactone [39–44]. Meager work has been performed investigating the effect of IH on sepsis or age relate chronic inflammatory conditions. Although meager research has been conducted in IH, the findings presented here are in alignment with existing researchers who have reported its capacity to attenuate iNOS/NO2-, COX-2/PGE2, HMGB1 release and NF-κB in LPS-activated RAW 264.7 cells or phorbol activated T cells [45–48]. Interestingly, although we did not find IH to have significant antimicrobial effects on *E.coli* 0157:H7 (1x10^6^ CFU/mL) at the low concentrations criteria used in this study, others have reported its capacity to destroy invasive pathogens such as *Staphylococcus aureus*/methicillin-resistant (MRSA) gram-positive bacteria, yeasts parasites and *Mycobacterium tuberculosis* [42, 49–53]. These studies suggest IH as being somewhat promising for attenuating inflammation arising from diverse infective or inflammatory insults.

The data from this work also show that fresh dried ethanol extracts of Bay leaf (BL) **(** *Laurus nobilis* ***)*** contains anti-inflammatory properties [54, 55]. Previous work by others demonstrates the oil (not aqueous) extracts to contain antimicrobial/food preserving properties due to cineole, eugenol, pinene, eucalyptol, linalool, carvacrol and α-terpinenyl acetate all evidentially toxic to Gram-positive bacteria (*Staphylococcus aureus/pyogenes*) and fungi (*Candida albicans, Aspergillus fumigatus*) [56–59]. Again, regarding the aqueous extract of BL, our work corroborates the work of others having reported the capacity to attenuate LPS mediated microglia/macrophage activation thought to be attributable to its sesquiterpene content [60, 61]. These type of substances are thought to be beneficial in chronic age related degeneration, by not only reducing inflammation but also blocking neurotoxicity of AD pathological Aβ (25-35)-component fragments [62].

Another herbal extract used in the current study to which little data exists is *Centipeda minima* (CM). CM has previously been reported to contain high levels of helenalin with the capacity to LPS mediated elevation of NO_2_–,TNF-α, IL-1b, iNOS and cyclooxygenase-2 in macrophages [63]. CM also demonstrates the capacity to attenuate tissue injury *in-vivo* involving inflammation such as carrageen paw edema and liver fibrosis [63–65]. Although we did not find CM to have significant antimicrobial effects on E.coli 0157:H7 (1 x 10^6^ CFU/mL) at the low concentration criteria used in this study, others have reported its capacity to kill *Enterobacter aerogenes*, *Staphylococcus aureus*, *Yersinia enterocolitica* and *Bacillus subtilis* [66, 67].

*Feverfew (Tanacetum parthenium)* (TP) is another rarely evaluated herb which long been reported to treat inflammatory conditions including psoriasis, allergies, arthritis, asthma and particularly migraines [68]. TP derived sesquiterpene lactones such as parthenolide are believed to be responsible for observed anti-inflammatory effects in animal models of carrageenan-induced edema, osteoarthritis, colitis cystic fibrosis and phorbol triggered mouse-ear edema [69–73]. TP constituents also antagonize toll-like receptors, Akt/mTOR and NF-κB pathways and block the downstream release of cytokines [74, 75]. Like the present study, previous research reports also corroborate capacity to reduce LPS activation of BV-2 cells and RAW 264.7 cells alike [76, 77]. There is also an antinociceptive aspect to feverfew commonly reported, having benefit to ameliorate pain associated with diabetic peripheral neuropathy [78].

The use of tansy (*Tanacetum vulgare*) (TV) as a medicinal plant was reported dating back to the 8th century A.D., when the Benedictine monks used it to treat intestinal worms, ameliorate digestive problems, fevers and sores. Interestingly, both feverfew and tansy have in common hyper allergenic potential due to parthenolide [79, 80]. Tansy is also rich in flavonoid glycosides, 7-O-glucosides of apigenin, luteolin, scutellarein and 6- hydroxyluteolin, chrysoeriol and eriodictyol as well as aglycones, hispidulin, nepetin, eupatilin, jaceosidin, pectolinarigenin and axillarin [81]. The oil contains 1,8-cineole and β-thujone as a major constituent along with carveol, umbellulone, davanone, dihydrocarvone, chrysanthenol, borneol and myrtenol [82–85].

Ashwagandha *(Withania somnifera)* (WS) is a highly studied herb with a plethora of known health benefits, in particular for prevention of cardiovascular disease. Its primary bioactive compound (Withaferin A) directly inhibits β1-adrenergic receptors, HMG-CoA, angiotensinogen-converting enzyme, total cholesterol, triglycerides, low density lipoprotein and elevation of protective high density lipoproteins and endogenous antioxidant systems [86, 87]. In animal models, WS prevents isoproterenol induced myocardial infarction, stroke distal middle cerebral artery occlusion and monocrotaline induced pulmonary hypertension in rats [88–91]. With respect to the immune system, WS can attenuate mitogen induced T/B-cell activation, secretion of Th1 and Th2 cytokines and inhibit NF-κB nuclear translocation in lymphocytes [92]. These immunomodulating effects of WS are also reported in-vitro for systemic LPS or *E. coli* administration in mammals, where there is a reduction in neutrophil tissue infiltration [93, 94] as well as tissue damage and pain associated with rheumatoid arthritis [95–97]. Ashwagandha is also an anti-infective agent lethal to gram-positive bacteria/cocci such as methicillin resistant *Staphylococcus aureus* and *Enterococcus*, respectively [98]. The data in this study ranks, validates and confirms pre-existing research showing significant antimicrobial effects of green tea EGCG polyphenon-60 (PP-60) *Acacia arabica*,grapeseed extract, caffeic, gallic acid, chapparal (*Larrea tridentata*) [99–109], where little has been investigated on antimicrobial herbs such as balm of Gilead Bud (*Populus candicans*), an herb of great historical significance with observed antibacterial and anti-inflammatory properties.

## Conclusion

In conclusion, the data obtained in this work affords general information on validated CAM anti-inflammatory and antimicrobial compounds and relative potency at sub lethal concentrations in LPS activated BV-2 and RAW 264.7 cells. Moreover, the data obtained also provide relative lethal potency of CAMs against the growth of *E.coli 0157:H7.* These findings can serve as a guide for future examination of specific CAM based herbal/nutraceutical anti-inflammatory/antimicrobial modalities for use in prevention or treatment of disease.

## Abbreviations

ANOVA: One-way analysis of variance
CAMs: Complementary and alternative medicines
CM: Centipeda minima
CNS: Central nervous system
COX2: Cyclooxygenase-2
DMEM: Dulbecco’s modified eagle medium
EGCG: (-)-epigallocatechin gallate
ELISA: Enzyme-linked immunosorbent assay
GCSF: Granulocyte-colony stimulating factor
HMGB1: High mobility group box 1 protein
HTP: High throughput
iEI: *in-vitro* efficacy index
IH: *Inula helenium*
INOS: Inducible nitric oxide
LPS: Lipopolysaccharide
MSRA: Methicillin-resistant
NO2-: Nitrite
NSAID: Non-steroidal anti-inflammatory drugs
PBS: phosphate buffered saline
RANTES: Regulated on activation, normal T cell expressed and secreted
TP: *Tanacetum parthenium (Feverfew)*
TV: *Tanacetum vulgare (Tansy)*
WS: *Withania somnifera (Ashwagandha)*
